# Outcomes following surgical repair of absent pulmonary valve syndrome: 30 years of experience from a Swedish tertiary referral centre

**DOI:** 10.1093/icvts/ivac193

**Published:** 2022-07-28

**Authors:** Vasileios Avdikos, Jens Johansson Ramgren, Katarina Hanséus, Torsten Malm, Petru Liuba

**Affiliations:** Cardiology, Pediatric Heart Center, Skåne University Hospital, Lund, Sweden; Cardiac Surgery, Pediatric Heart Center, Skåne University Hospital, Lund, Sweden; Cardiology, Pediatric Heart Center, Skåne University Hospital, Lund, Sweden; Cardiac Surgery, Pediatric Heart Center, Skåne University Hospital, Lund, Sweden; Cardiology, Pediatric Heart Center, Skåne University Hospital, Lund, Sweden; Clinical Science, Lund University, Lund, Sweden

**Keywords:** Absent pulmonary valve syndrome, Surgical repair, Respiratory distress, Outcome

## Abstract

**OBJECTIVES:**

Absent pulmonary valve syndrome is a rare congenital heart defect with pulmonary artery dilatation and secondary airway compression. Although preoperative respiratory support and early surgical repair with pulmonary arterioplasty are often required in patients with airway compromise, the need for extensive plasty in these patients and for plasty in general in those with no or mild respiratory issues remains debatable.

**METHODS:**

We performed a retrospective survey of patients with this diagnosis and repair from 1988 to 2018.

**RESULTS:**

Twenty patients were identified. The median age and weight at repair were 0.8 (0.1–2.4) years and 7.0 (2.5–13.8) kg and included a valved conduit in 17 (85%) patients and a transannular patch in 3 patients. Five (29%) patients were ventilator-dependent prior to repair at the age of 0.3 (0.1–0.4) years. Pulmonary arterioplasty was performed in 7 patients (35%), including all 5 with ventilator dependency and 2 with respiratory symptoms due to recurrent infections. Two patients (10%) with preoperative ventilator dependency underwent extensive intrahilar arterioplasty. Preoperative ventilator dependency was associated with earlier repair and reinterventions (*P* < 0.05). There were 3 late deaths among cases with repair after 2000 (n = 14), none with preoperative ventilator dependency.

**CONCLUSIONS:**

The long-term outcomes of patients with this rare defect are good, comparable to those of other previous studies. Reduction pulmonary arterioplasty, which in this study was used only in patients with respiratory distress and ventilator dependency, is associated with excellent survival. Reinterventions are common in these patients.

**Figure:**
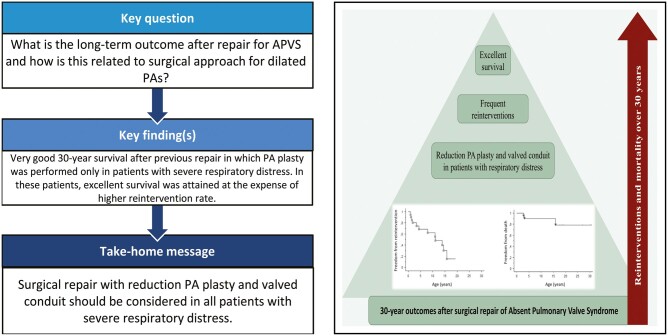
Surgical approach with reduction pulmonary artery plasty and valved conduit in patients with respiratory compromise prior to repair is associated with excellent long-term survival at the cost of a higher reintervention rate.

## INTRODUCTION

Absent pulmonary valve syndrome (APVS) is a rare congenital heart disease first described by Chevers in 1847 [1]. It shares some features with tetralogy of Fallot (ToF); it is observed in 15–20% of fetuses and 3–6% of neonates with ToF [2, 3]. APVS includes a malalignment type of ventricular septal defect (VSD), various degrees of pulmonary annular stenosis, rudimentary or absent pulmonary valve and free pulmonary regurgitation. The landmark is the aneurysmal dilatation of the main pulmonary artery (PA) and its branches. This condition can lead to compression of the tracheobronchial tree and subsequent respiratory compromise [2]. Neonates and infants may present with severe respiratory problems or signs of cardiac decompensation requiring preoperative mechanical ventilation (MV) and urgent surgical repair with varying degrees of PA plasty ranging from a moderate reduction of the size of the PAs to extensive intrahilar reduction. They also tend to have a more complex postoperative course with respiratory complications, continued ventilator dependency, multiple hospitalizations for recurrent respiratory infections secondary to tracheobronchomalacia and air trapping and worse survival compared with infants with minimal or no airway symptoms. In the latter, the clinical phenotype resembles that of ToF, and the surgical repair is typically delayed to the age of 6 to12 months, because it was shown to be associated with better outcomes in terms of postoperative and long-term morbidity [4–7]. With regard to the surgical approach for dilated pulmonary arteries, the need for PA reduction in this group of patients with stable respiratory conditions remains debatable.

The survival of patients with APVS has improved markedly with a decrease in postoperative mortality from 40–60% in the 1960s and 1970s [2, 8] to 10–20% in recent years [9–13], with most deaths occurring among neonates and infants with severe respiratory distress. Several studies [11, 12, 14] have identified preoperative ventilator dependency as a significant risk factor for poor outcomes and high mortality in this age group.

Despite improvement in survival, patients with APVS have high reoperation rates, and many require multiple reinterventions on the right ventricular outflow tract (RVOT) as well as reoperations of PAs due to persistent respiratory problems postoperatively [7, 11, 14–16].

Owing to the rarity of APVS, few studies on long-term outcomes have been published so far. The goal of this retrospective study was to assess survival and the need for reinterventions over the past 30 years of APVS treatment with particular focus on the use and extent of PA plasty in infants with stable respiratory conditions and in those with severe airway compromise prior to repair.

## MATERIAL AND METHODS

### Patients

We included all consecutive patients referred for surgical repair of APVS in Lund from 1988 to 2018. Since 1993, Lund has been a tertiary referral centre for paediatric congenital heart surgery, serving half of the Swedish population.

One female patient died prior to surgery and was excluded from the study. She presented early after birth with symptoms of severe respiratory distress requiring MV. She remained in the intensive care unit (ICU) until the age of 1.5 months when the decision was made to discontinue treatment due to clinical deterioration. Prior to this decision, magnetic resonance imaging showed further dilatation of the PAs with significant compression of the tracheobronchial tree. A post-mortem examination revealed significant narrowing of the tracheal lumen proximally to the pulmonary bifurcation, widespread areas of lung emphysema with non-functional parenchyma and significant septal and myocardial hypertrophy without signs of myocardial infarction.

Clinical records of all patients with APVS who underwent surgical correction at our institution were reviewed. Medical data were retrieved from the paper-based medical records available in the hospital archives for patients born from 1988 through 2000 and from the electronic patient chart system for patients born after 2001.

There were no missing data for the variables included in the study.

### Definitions

Urgent repair was defined as repair due to ventilator dependency.

Reintervention included reoperation or therapeutic catheterization.

Early mortality was defined as death occurring during the first 30 days after surgery or before hospital discharge. All other deaths were considered late deaths.

### Data analysis

Statistical analyses were performed using StatView Version 5.0 (SAS Institute, Cary, NC, USA). Data for all continuous variables are shown as median and range. Categorical variables are expressed as frequencies and percentages. The non-parametric Mann-Whitney U test was used to assess the differences between patients with and without preoperative MV based on their age at surgery, cardiopulmonary bypass time, aortic cross-clamp time (CCT), postoperative ICU and total length of stay (LOS), age at reintervention and number of reinterventions. We also compared these 2 subgroups for deaths and reinterventions (outcome variables) using the Kaplan-Meier curve. Differences in survival distribution were identified using the log-rank test.

The following variables were included in the univariable analysis: age and weight at surgery, cardiopulmonary bypass time, aortic cross-clamp time, preoperative MV, ICU LOS, fetal diagnosis, presence of chromosomal abnormalities, additional CHD, right ventricular systolic pressure measured invasively in the operating room after total repair, postoperative MV (yes/no) and length, postoperative ICU LOS, total postoperative LOS, elective versus urgent repair, reintervention (reoperation or transcatheter intervention) and overall number of deaths. A multivariable analysis was deemed not suitable due to the small sample size. Two-tailed *P* < 0.05 was considered statistically significant.

## RESULTS

### Demographics

We identified 20 patients (11 female; 55%) with surgical repair for APVS between 1988 and 2018. The median age and weight at repair were 0.8 (0.1–2.4) years and 7.0 (2.5–13.8) kg, respectively. Two patients (10%) were born prematurely, both at 32 weeks of gestation.

The diagnosis of APVS was made prenatally in 6 patients (30%). Postnatally, it was made due to cardiac murmur (*n* = 6), cyanosis (*n* = 2), respiratory problems (*n* = 4) or multiple malformations (*n* = 2).

### Associated intracardiac and extracardiac anomalies

All patients had associated ToF. Other associated cardiovascular anomalies included atrial septal defect (*n* = 4; 20%), right aortic arch (*n* = 7; 35%), major aortopulmonary collateral arteries (MAPCA) (*n* = 2; 10%), non-confluent PAs with significant hypoplasia of the left pulmonary artery (LPA) (*n* = 1; 5%), dextroposition of the heart (*n* = 1; 5%) and persistent left superior vena cava (*n* = 2; 10%). No patients had coronary artery anomalies. Of the 5 patients screened for 22q11 syndrome, 3 (15%) had positive test results. An additional 2 patients (10%) had phenotypic features of 22q11 but the parents denied chromosomal analysis. One patient (5%) had VACTERL (vertebral defects, anal atresia, cardiac defects, tracheo-oesophageal fistula, renal abnormalities and limb abnormalities) syndrome; 1 (5%) had CHARGE (coloboma, heart defects, atresia choanae, growth retardation and ear abnormalities) syndrome; and 1 (5%) had Alagille syndrome.

### Prerepair care

Five (25%) patients (1 neonate and 4 infants) required preoperative respiratory support with MV due to severe airway compression (Tables 1, 2). One of the 2 additional patients with respiratory distress due to recurrent respiratory infection required non-invasive ventilation (Table 2).

**Table 1:** Demographic and surgical characteristics in the whole cohort and in the subgroups of ptients with and without mechanical ventilation prior to repair

	Median (range) or number (%)		
Variables	Total	MV group	Non-MV group
	(n = 20)	(n = 5)	(n = 15)
Number of patients with cardiology comorbidities	17 (85)	4/5 (80)	7/15 (47) *
Preoperative ICU length of stay	0 (0-41)	6 (4-41)	0 (0-0)*
Preoperative MV time (days)	0 (0-27)	6 (4-27)	0 (0-0)*
Age at surgical repair (years)	0.8 (0.1-2.4)	0.25 (0.1-0.4)	1.5 (0.1-2.4)*
Weight at surgical repair (kg)	7.0 (2.5-13.8)	4.1 (2.8-6.7)	8.1 (2.5-13.8)*
Cardiopulmonary bypass time (min)	152 (92-221)	178(157-221)	147 (92-202)*
Aortic cross-clamp time (min)	62 (5-142)	107 (67-142)	49 (5-90)*
Right ventricular systolic pressure (mmHg)	31 (19-44)	25 (20-41)	31 (19-44)
Systemic arterial pressure (mmHg)	78 (52-102)	78 (52-87)	78 (62-102)
Emergency repair	7 (35)	5/5(100)	2/15 (13)*
Reduction pulmonary arterioplasty	7 (35)	5/5 (100)	2/15 (13)*
**Types of RVOT reconstruction**			
Valved conduit	17 (85)	5/5 (100)	12/15 (80)
Pulmonary homograft	4 (20)	1/5 (20)	3/15 (20)
Aortic homograft	6 (30)	1/5 (20)	5/15 (33)
Contegra bovine jugular vein valved conduit	7 (35)	3/5 (60)	4/15 (27)*
Transannular patch	3 (15)	0/5 (0)	3/15 (20)*

* Denotes *P* < 0.05 (MV group vs non-MV group compared with the Mann-Whitney test).

ICU: intensive care unit; MV: mechanical ventilation; RVOT: right ventricular outflow tract.

**Table 2:** Characteristics of patients who underwent reduction pulmonary arterioplasty

Patient number	Gender	Preoperative MV (Yes/No)	Clinical symptoms	Preoperative CT/MRT findings	Age at repair (years)	Types of reduction PA plasty	Outcome
1	Female	No	Recurrent respiratory tract infections with significant respiratory symptoms	Significant dilatation of MPA, proximal RPA and LPA with flattening of the left main bronchus from the aneurysmal MPA; no compression of the right main bronchus	0.40	Anterior resection of MPA	Well at follow-up
2	Female	Yes (21 days)	Frequent respiratory tract infections, on NIV	Massively dilated PAs, particularly RPA (20 mm) with compression of the trachea and both mainstem bronchi from the aneurysmal RPA	0.40	Anterior resection of RPA	Well at follow-up
3	Male	Yes (4 days)	Severe respiratory distress	Aneurysmal PAs > 15 mm, particularly RPA(20 mm), compression of right main and upper lobe bronchi	0.25	Anterior and posterior resection of RPA, LPA, Lecompte manoeuvre	Well at follow-up
4	Male	No	Frequent respiratory tract infections	Significant dilatation of MPA(17 mm), RPA(7 mm), LPA(9 mm), significant compression of the left main bronchus after tracheal carina between the descending aorta and the massively dilated MPA	0.30	Anterior and posterior resection of MPA, stretching of RPA, LPA	Well at follow-up
5	Female	Yes (6 days)	Severe respiratory distress, need for ventilatory support from birth	Aneurysmal PAs, RPA(15 mm), LPA(20 mm), distal stenosis of MPA, significant bronchial compression	0.10	Extended anterior and posterior resection of RPA, LPA to the hilum, Lecompte manoeuvre	Well at follow-up (BiPAP at night)
6	Male	Yes (27 days)	Severe respiratory symptoms, difficulties in weaning from ventilator	Massively dilated PAs, RPA(16 mm), LPA(17 mm), mild stenosis of distal trachea and proximal right main bronchus, significant compression of the left main bronchus	0.20	Anterior and posterior resection and plication of RPA, LPA	Well at follow-up
7	Female	Yes (6 days)	Symptoms of heart failure, cyanosis, stridor	Significant dilatation of MPA(20 mm), RPA(15 mm), LPA(14 mm), significant bilateral main bronchial compression	0.40	Extended anterior and posterior resection and plication of MPA, RPA, LPA to the hilum, arteriopexy of RPA, LPA, suspension of PA to the retrosternal fascia	Well at follow-up

BiPAP: bilevel positive airway pressure; LPA: left pulmonary artery; MPA: main pulmonary artery; MV: mechanical ventilation; NIV: non/invasive ventilation; PAr: pulmonary artery reduction; RPA: right pulmonary artery.

Five patients (25%) had 1 cardiac and 3 non-cardiac interventions before repair. Of those with cardiac interventions, 1 (male) had disconnected PA branches and significant hypoplasia of the LPA. This patient initially had a right modified Blalock-Taussig shunt (mBTs) at 2 days, a left mBTs at 3 months and repair at 2.4 years of age. Another patient (female) had coil embolization of 3 major aortopulmonary collateral arterial vessels 4 days prior to repair at 1.9 years.

Of those with non-cardiac interventions, 1 patient (female) had a right lower lobectomy due to pulmonary sequestration at 16 months with repair at 2 years. A second patient (female), with VACTERL (vertebral defects, anal atresia, cardiac defects, tracheo-oesophageal fistula, renal abnormalities and limb abnormalities) syndrome had repairs of anal atresia and lip-palate cleft at 2 weeks and 9 months of age, respectively, with APVS repair at 1.5 years. A third patient (female, preterm) was operated on twice for bilateral choanal atresia at 4 days and 3 months and had a repair at 1.5 years.

A computed tomography scan or a magnetic resonance imaging scan was performed on all patients as part of our routine preoperative imaging protocol. None had preoperative assessment with fibre-optic bronchoscopy.

### Surgical repair

The surgical data for all patients and for patients with and without preoperative MV are summarized in Table 1.

All 5 patients (1 neonate and 4 infants) with preoperative MV underwent urgent repair with an RVOT conduit (Table 1) from 1 week to 4 months of life, while the remaining patients had conduit or non-conduit repair (Table 1) on an elective basis. The majority of patients (n = 11, 55%) had repair during the first year of life. All but 1 patient, who was born before 1990 and was first palliated with a right mBTs at day 2 followed by repair at 2.4 years of age, had a single-stage repair.

Patients with preoperative MV had longer cross-clamp times (*P* = 0.02) and longer cardiopulmonary bypass times (*P* = 0.04) than the non-MV patients (Table 1).

### Reduction pulmonary plasty during surgical repair

The characteristics of patients in whom PA plasty was performed are outlined in Table 2. PA plasty was first performed at our centre in 2006. Seven (35%) patients, all < 1 year of age including 5 with preoperative MV and 2 with multiple hospitalizations due to recurrent respiratory infections, had this procedure. Among the patients without PA plasty at repair (*n* = 13), 4 patients with repair before 1 year of age had in the neonatal period respiratory problems that required MV or continuous positive airway pressure due to bronchial compression, but these issues resolved over time. For this reason, PA plasty was not performed. Similarly, none of the patients with repair after 1 year of age (*n* = 9) had significant respiratory problems at the time of the surgery, but some had earlier signs of tracheobronchial compression that improved over time.

In addition to reduction PA plasty, 2 (10%) patients underwent the Lecompte manoeuvre in order to displace the dilated PA away from the trachea and bronchial tree.

### Postoperative outcome

The postoperative data are summarized in Table 3. The postoperative course was uneventful in 12 patients (5 infants and 7 children). In all patients with preoperative MV (n = 5), the postoperative course was complicated by persistent respiratory problems, air-trapping due to bronchomalacia, desaturation, paralysis of the left diaphragm or secondary infections. However, all but 1 (who still needs bilevel positive airway pressure at night) recovered fully and did well at follow-up (Table 2).

**Table 3:** Postoperative data of patients with absent pulmonary valve syndrome

	Median (range) or number (%)		
Postoperative data	Total (n = 20)	MV group (n = 5)	Non-MV group (n = 15)
Postoperative ICU LOS (days)	3 (1-68)	18 (6-68)	3 (1-5)^*^
Postoperative MV length (days)	1 (0.4-21)	13 (1-21)	1 (0.4-2)^*^
Total in-hospital LOS (days)	14.5 (7-151)	46 (23-151)	14 (7-82)^*^
Early mortality	0 (0)	0 (0)	0 (0)
Late mortality	3 (15)	0 (0)	3 (20)
Reintervention (surgical or transcatheter)	12 (60)	4 (80)	8 (53)^*^
Age at first reintervention (year)	8 (0.9-19.3)	1.8 (1.1-5.3)	11.1 (0.9-19.3)^*^
Surgical reintervention	10 (50)	4 (80)	6 (40)^*^
Transcatheter intervention	4 (20)	1 (20)	3 (20)
Second reintervention	3 (15)	1 (20)	2 (13)

* Denotes *P* < 0.05 (MV group versus non-MV group compared using the Mann-Whitney test).

ICU: intensive care unit; LOS: length of stay; MV: mechanical ventilation.

In the non-MV group, complications included short-term convulsions (*n* = 1), pneumothorax requiring a chest-tube drain (*n* = 1) and residual VSD (*n* = 1).

One of the patients in the MV group required a tracheostomy on day 22 postoperatively due to perioperative respiratory complications with left diaphragm paralysis and difficulties in weaning from MV. This patient had surgical repair with extensive reduction of the PAs from hilum to hilum as well as the Lecompte manoeuvre. During the first postoperative year, the patient remained dependent on the ventilator but improved thereafter and currently required bilevel positive airway pressure only at night.

### Reinterventions

**Figure 1:**
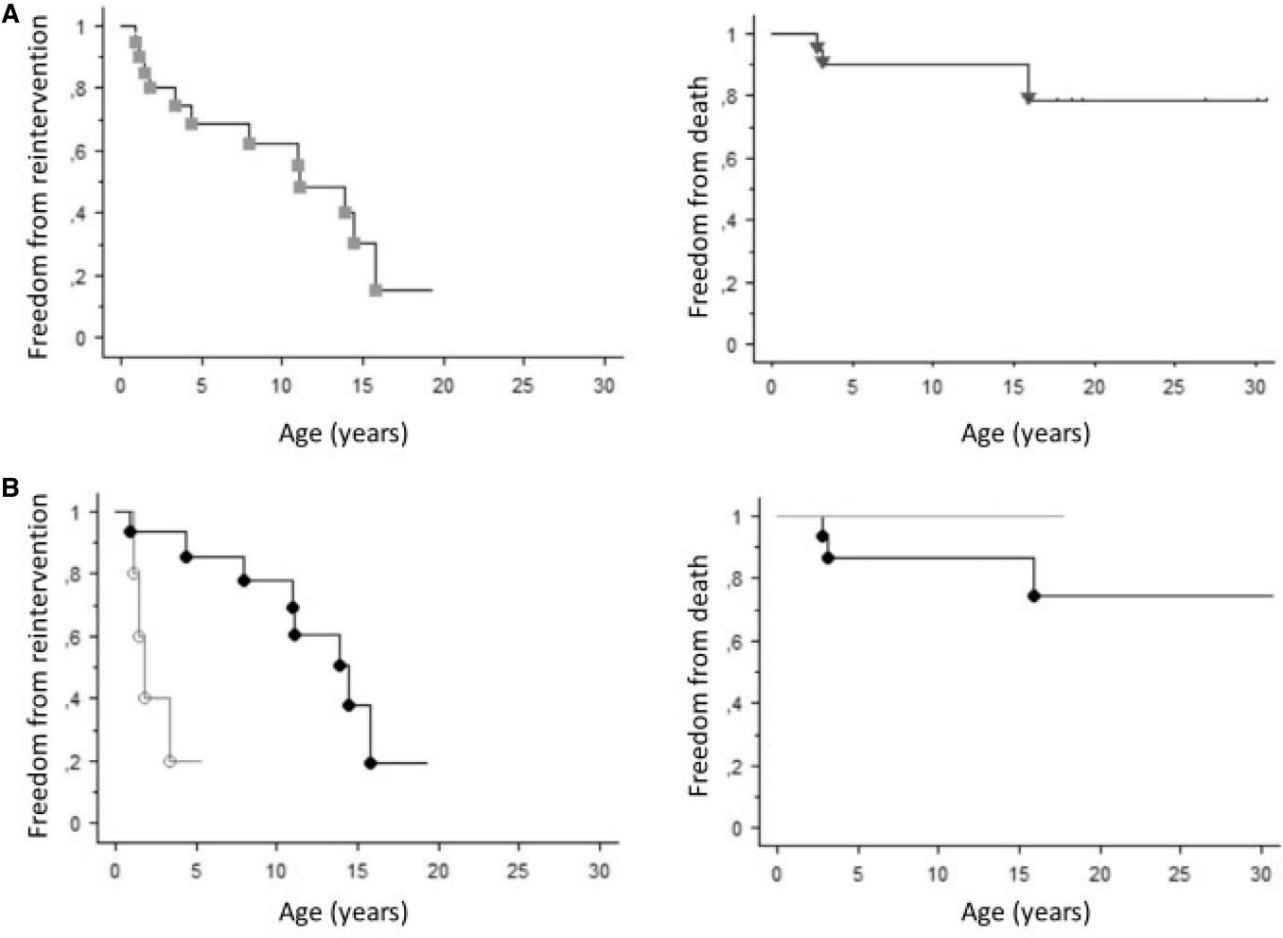
Freedom from surgical or transcatheter reintervention (left) and from death (right) in all patients (Panel A) and in patients with (dark-grey line) and without (black line) mechanical ventilation (MV) prior to surgical repair (Panel B). Log-rank *P* < 0.01 for the MV group versus the non-MV group, both for freedom from death and freedom from reinterventions.

Freedom from reintervention (surgical or transcatheter) in the entire cohort is shown in Figure 1/Panel A (left). Altogether, reinterventions were needed in 12 patients. Of these, 3 (15%) patients required > 1 reintervention. The first reinterventions were performed in 12 (60%) patients at the median age of 6.2 (0.9–15.8) years. The time interval between repair and the first reintervention was 4.9 (0.7–13.9) years.

Patients with MV prior to repair had earlier and more reinterventions (conduit replacement with or without the need for repeated PA plasty and pulmonary valve replacement) compared with the other patients (Table 3 and Figure 1/Panel B; *P* < 0.05). Surgical reintervention was required in 10 patients (50%) at the age of 5.6 (0.9–13.9) years and included conduit replacement in 9 patients and a conduit implanted in 1 patient. Three of the patients with conduit replacement belonged to the MV group and underwent repeated reduction PA plasty along with conduit replacement. The patient with a conduit implant had a repair with a transannular patch (TAP) at the age of 0.7 years and conduit surgery 10.4 years later. Of the other 2 patients repaired with a TAP, 1 died 2.4 years later of an acute myocardial infarction (detailed under Mortality). The third patient, who underwent repair at 0.4 months of age, has not required reintervention thus far (current age 19.3 years).

Transcatheter intervention was performed in 4 patients at the age of 13.8 (4.4–15.8) years and included pulmonary valve replacement with a Melody valve (Medtronic, Minneapolis, MN, USA) in 3 patients and an LPA stent in 1 patient.

One infant with preoperative ventilatory support prior to surgical repair, despite extensive mobilization and reduction of PAs from hilum to hilum at the time of the primary repair, had persistent respiratory distress due to compression of the airways by dilated PAs. She underwent a reoperation 7 days later with arteriopexy of PAs and suspension of the PA to retrosternal fascia with successful improvement of respiratory problems.

A second reintervention was required in 3 (15%) patients; it included conduit replacement in 2 patients and an LPA stent in 1 patient. The latter had a primary repair at 0.3 years of age including stretching of the PAs to alleviate the compression of the left main bronchus caused by the aorta and the dilated PAs. This patient had recurrent infections from the left lung and underwent partial lobectomy of the left upper lobe at the age of 1.6 years with successful improvement of the respiratory symptoms.

### Mortality

Freedom from mortality in the entire cohort and in patients with and without MV prior to repair is shown in Figure 1, Panels A and B. There were no early deaths. There were 3 later deaths (15%), occurring at 5 months, 2.4 and 13.9 years, respectively, after surgical repair. The median age at death was 3.1 (2.5–15.9) years. None of these 3 cases had required preoperative MV. There were no deaths among patients who had surgical repairs after 2000 (*n* = 14).

The first death occurred in the early 1990s of significant pulmonary hypertension. This patient was initially palliated with a right mBTs at the age of 2 days and underwent surgical repair at the age of 2.4 years with failure to completely close the VSD. The post-mortem histopathological examination revealed moderate intimal thickening of the pulmonary arteries of the right lung as well as signs of acute bronchitis, oedema and significant interstitial fibrosis of the left lung.

The second patient was a premature boy born in the 32nd week who had surgical repair with a TAP at 0.7 years of age and who died at 3.1 years of age. He presented to the emergency room with acute chest pain, pronounced paleness, convulsions and bradycardia with no response to cardiopulmonary resuscitation and medications. The post-mortem examination showed areas with suspected fibrosis in the myocardium of the left ventricle but no signs of coronary abnormalities. Histopathological examination showed areas of acute myocardial infarction with slight degeneration of muscle fibres, interstitial oedema and granulocytes.

For the third patient (female), the repair was done at the age of 2 years with an 18 -mm aortic homograft; a Melody valve was implanted percutaneously at the age of 14 years, 1.5 years before her death. The autopsy did not reveal any clear cause of death, with normal coronaries and cardiac and pulmonary morphology.

## DISCUSSION

This study summarizes our 30-year experience managing patients undergoing surgical repair of APVS at a Swedish tertiary paediatric congenital heart disease centre. The findings indicate that the long-term outcome is good, with 3/20 (15%) deaths (the 3 patients who died were operated on before the year 2000) and no deaths among the 7 patients (35%) with compromised airways. Importantly, PA plasty has been used at our institution only in the latter group and in combination with the valved conduit, thus reconfirming the necessity of PA reconstruction to alleviate tracheobronchial compression in order to improve survival in these high-risk patients. Six of the 7 patients with airway compromise had repair with PA plasty after 1 month of age, possibly suggesting that delayed repair beyond the neonatal period should be attempted whenever possible. Further studies are needed to confirm this.

During the past decades, improvements in surgical techniques, better understanding of the underlying pathology and advances in postoperative care and ventilatory management of neonates and infants have led to improved survival among these patients. The overall survival of 85% is consistent with the results noted in previously published studies reporting a 10-year survival of approximately 80% [9–13].

Multiple previous studies [7, 11, 12, 14, 15] have identified preoperative intubation and ventilation as a risk factor for early postoperative complications. Airway compression by the aneurysmal PAs is seemingly an important underlying mechanism [2, 4]. McDonell *et al.* [7] reported an early mortality of 21% (6/28, all infants with preoperative ventilator dependency), whereas Yong *et al.* [11] recently reported a lower early mortality of 13.5% (7/52, 15 with preoperative MV, all under 1 year of age). In our institution there were no early deaths among neonates and infants (11/20), although 5 patients (1 neonate and 4 infants) needed MV prior to surgery and underwent urgent repair. One of them required a tracheostomy postoperatively due to paralysis of the left diaphragm followed by gradual recovery. All of them are doing well in the follow-up period.

In our study, preoperative ventilator dependency was associated with longer postoperative and total LOS. These patients often have a complicated course due to persistent respiratory symptoms and secondary infections postoperatively. These factors are attributable to the underlying tracheobronchomalacia caused by external compression attributable to the aneurysmal PAs [13, 15, 16]. Persistent bronchomalacia requires prolonged duration of MV, which also increases the risk for pulmonary infections, thus leading to a longer postoperative LOS. We also found that patients with preoperative MV need reintervention at an earlier age. This need is most likely due to the fact that the primary repair occurred at a younger age, with the majority of patients (85%) receiving a valved conduit of a smaller size and thus one would expect failure with increasing age. Similarly, Alsoufi and colleagues [14] found younger age at the time of conduit placement to be a risk factor for reoperation.

Different surgical options have been proposed regarding RVOT reconstruction, such as valved conduit, homograft or TAP with or without a monocusp valved patch. There is still no consensus about the best method to restore RV-PA continuity in patients with APVS. Some centres recommend insertion of a valved conduit in order to improve the early postoperative haemodynamics, avoid persistent PA dilatation and reduce the risk of long-term arrythmias and late right ventricular dysfunction [7, 14–17, 19]. Others consider that TAP with or without a monocusp valve is sufficient [6, 9, 10, 17, 18]. Godart and associates [6] advocate for insertion of a pulmonary valve only in severely ill neonates with high pulmonary vascular resistance or distal pulmonary stenosis. Regardless of the preferred method for establishing an RV-PA connection, no difference was found in the overall mortality or risk for reoperation [11, 12, 14]. In our institution, TAP was used in only 3 patients, all older than 3 months at repair. We believe that insertion of a valved conduit is extremely important for patients requiring early repair in order to eliminate pulmonary valve regurgitation and the volume load of the RV. Since 2002, it has been our practice to use valved conduits whenever possible.

The majority of patients with previous repair require multiple RVOT reoperations [7, 11, 14, 16], which is consistent with our findings. Yong and associates [11] reported freedom from reoperation of 79.7% ± 6.9% at 5 years and 69.4% ± 8.2% at 10 years, respectively. Alsoufi and co-workers [14] reported freedom from reoperation of 89% ± 5% and 59% ± 9% at 5 and 10 years. Dodge-Khatami and associates [16] reported a reoperation rate of 40% (4/10; mean follow-up, 25.5 months).

Various surgical techniques have been used over time to reduce the tracheobronchial compression caused by the severe dilatation of PAs, with variable results. Those methods included the use of the Glenn shunt, anterior and posterior plication of the aneurysmal PAs, reduction of the PA by excising parts of the anterior or posterior walls, translocation of the PA anterior to the aorta with the Lecompte manoeuvre, complete replacement of the entire PA with placement of a bifurcated pulmonary homograft or suspension of the PA to the retrosternal fascia [7–9, 17–20]. Endobronchial stents have been recommended in order to treat the persistent tracheobronchomalacia and respiratory problems in those patients who could not be weaned from MV [16, 21]. The downsides include limited stent dilatation and lack of absorbable material with the need for lifelong follow-up with bronchoscopy [21]. No such stents were used in our centre. The Lecompte manoeuvre was used in 2 of our patients with bilateral bronchial compression, combined with anterior/posterior wall excision; there were no deaths.

Although many centres recommend extensive reconstruction of the PAs in patients with severe respiratory distress [9, 11, 17, 18], in our centre, the decision as well as the optimal method for reduction PA plasty is individualized, depending on the severity of the respiratory symptoms, the degree of PA dilatation and the presence of compression of the tracheobronchial tree in the preoperative computed tomography/magnetic resonance images. Thus, if there are no significant symptoms related to airway compression, reduction PA plasty is not performed due to the expected decrease in pulmonary pressure and the subsequent wall tension after repair. The benefit of this approach is arguable given the fact that all 3 deaths in our study (all before 2000) occurred in patients without preoperative significant respiratory issues who had repair without PA reduction, one of whom was repaired with TAP. In our cohort, reduction PA plasty was done in all patients with ventilator dependency or signs of severe cardiorespiratory distress (*n* = 7, all < 1 year of age) with no deaths. Only 2 of these patients (# 5 and # 7 in Table 2) underwent extensive PA reduction, suggesting that less extensive PA plasty strategies can be considered in some patients with respiratory distress. Extensive PA reduction, usually in combination with the Lecompte manoeuvre, is performed only in the presence of significant bilateral bronchial compression. Even if tracheobronchial compression is present in neonates and infants, improvement can be seen towards the end of the first year. This outcome is attributable to several factors, such as the change from cartilage to firmer bronchi, including the growth of the bronchi with the increase of the lumen [22].

### Study limitations

Due to its retrospective nature, selection bias and misclassification or information bias may have affected the interpretation of the data. Due to the rarity of APVS, there are a relatively small number of patients with a small number in each group, making it difficult to obtain meaningful comparisons. Also, the small sample size prevented the identification of factors related to poor outcomes in the follow-up period. For the same reason, a competing risk analysis between death and reintervention was not done. Changes over time in pre- and postoperative intensive care, different surgical techniques and skills are also important biases.

## CONCLUSION

The findings obtained in a relatively small cohort of patients with APVS repair during the past 30 years indicate that reduction PA plasty and valve insertion are paramount in patients with significant preoperative respiratory issues in order to improve long-term survival. As expected, reinterventions in this group of patients are common. Due to the rare nature and wide spectrum of severity of APVS, future multicentre studies would be able to overcome cohort size limitation and better answer the questions regarding the indication and extent of reduction PA plasty in this population.

## Funding

The study was funded by Lund University, the Swedish Children's Heart Association and the Swedish Heart-Lung Foundation.

## Data Availability

All relevant data are contained within the manuscript.

## Notes

Meeting presentation: 54th Annual Meeting of the Association for European Pediatric and Congenital Cardiology, 2021, Gothenburg, Sweden

## Abbreviations and acronyms

APVS: Absent pulmonary valve syndrome
ICU: Intensive care unit
LOS: Length of stay
MV: Mechanical ventilation
PA: Pulmonary artery
RVOT: Right ventricular outflow tract
TAP: Transannular patch
ToF: Tetralogy of Fallot
VACTERL: Vertebral defects, anal atresia, cardiac defects, tracheo-esophageal fistula, renal abnormalities and limb abnormalities
